# A Cross‐Organisational Collaboration to Promote Healthy Eating and Active Living in Children: A Critical Reflection

**DOI:** 10.1002/hpja.70063

**Published:** 2025-06-30

**Authors:** Sarah T. Ryan, Jennifer Norman

**Affiliations:** ^1^ School of Social Sciences, Faculty of the Arts, Social Sciences and Humanities University of Wollongong Wollongong New South Wales Australia; ^2^ Early Start University of Wollongong Wollongong New South Wales Australia; ^3^ Illawarra Shoalhaven Local Health District, Health Promotion Service Warrawong New South Wales Australia

**Keywords:** active living, collaboration, health promotion, healthy eating, systems approach

## Abstract

**Objectives:**

To critically reflect on a collaborative research partnership between university‐based researchers, a state government‐funded health promotion workforce, and policy makers, focused on promoting healthy eating and active living for children funded by the New South Wales (NSW) Health Prevention Research Support Program (PRSP).

**Importance of Study:**

Government bodies and academic institutions frequently operate in silos, creating knowledge translation challenges. Research takes time to reach practitioners, hindering uptake of evidence‐based interventions in public health settings.

**Study Type:**

Qualitative reflective evaluation.

**Methods:**

Thirty key stakeholders from the PRSP funded ‘EnHANCE’ research group collaboration were invited to complete an anonymous online survey. Open‐ended questions were structured around the six themes of Gibbs' Reflective Cycle (1998), to allow participants to reflect on their collaborative experiences spanning July 2017 to June 2022. Participants included University of Wollongong academics, PhD candidates, and NSW Health staff (managers and health promotion officers). Data were analysed using a deductive thematic analysis process, systematically identifying, analysing, and grouping into themes to highlight both the challenges and successes experienced by participants.

**Results:**

Fifteen participants responded. Challenges included COVID‐19 disruptions, navigating jurisdictional diversity, and initial misalignment of organisational priorities. Notable successes emerged, including the establishment of a strong, equitable research partnership, timely local implementation of research findings, enhanced cross‐jurisdictional learning and gradual alignment of research and health promotion priorities as relationships strengthened. A critical factor for the success of this collaboration has been the successive PRSP funding rounds which have given partnerships time to mature and be productive.

**Conclusions:**

The PRSP demonstrates an effective funding model for facilitating meaningful collaboration between academics, policymakers, and practitioners. Future funding schemes should aim to include opportunities to build strong collaborations, and researchers should integrate and explore adaptive, responsive models of cross‐organisational research partnerships.

## 1 Introduction

Historically, academics and policymakers have recognised the importance of generating research evidence to support healthcare policy and practice, yet government bodies and academic institutions often work in silos despite sharing common goals. This separation often results in research findings taking considerable time to filter down to practitioners on the ground, creating a knowledge translation challenge [1].

The Prevention Research Support Program (PRSP) is a funding initiative overseen by the New South Wales (NSW) Ministry of Health which provides opportunities for bridging gaps between academic research and preventive health research in NSW, Australia [2]. NSW Health, a state government body, funds and oversees health program delivery across 15 socially and geographically diverse jurisdictions, called local health districts [3]. These jurisdictions are responsible for delivering comprehensive health promotion programs to their communities, including supporting healthy eating and active living in children [4].

The PRSP provides funding to NSW research organisations to conduct prevention and early intervention research that aligns with NSW Health priorities [2]. The programme supports research infrastructure and strategies to build research capability within local health districts and to translate evidence from research into policy and practice.

Under Round 5 of the PRSP, Early Start at the University of Wollongong (UOW) was awarded AU$1.25 million from July 2017 to June 2022, in partnership with three NSW Health partners: South Western Sydney Local Health District (SWSLHD), Illawarra Shoalhaven Local Health District (ISLHD), and the Centre for Population Health (CPH). The Centre for Population Health leads the development, implementation and evaluation of policies, and funds evidence‐based programs and services to improve health and reduce the burden of chronic disease across all local health districts in NSW. The research group established though the PRSP (Round 5) funding is known as Evidence‐based Healthy Activity and Nutrition for Children and Environments (EnHANCE) [5].

The EnHANCE research group operates under a structured governance framework with two distinct levels of oversight. At the project level, monthly working groups were established for each project funded under Round 5. These groups included the Principal Investigator, Project Manager, UOW academics, PhD candidates (where applicable), and representatives from partner local health districts. Each local health district provided representation through both management and health promotion officers, ensuring balanced input from strategic planning and community implementation perspectives. The Centre for Population Health representatives were also integral working group members adding input from a state‐wide policy level perspective.

A Governance Committee was established to provide oversight of the entire EnHANCE research program. This Committee, which met quarterly under the leadership of the Principal Investigator and Project Manager, focused on accountability, strategy, risk management, and ensuring alignment with NSW Health priorities, particularly regarding research translation. Its membership included representatives from each partner local health district, the manager of Early Childhood and School Settings from the Centre for Population Health, and external experts in children's education and care settings and children's health research.

A subsequent round of PRSP funding (Round 6) is currently operating from July 2022 to June 2026, involving Early Start and the Round 5 partners, along with two additional local health districts (Western Sydney LHD and Southern NSW LHD), successfully securing a further AU$1.2 million in funding [1].

As a key goal of the PRSP funding is to support collaboration between NSW Health staff and academic researchers, there is a valuable opportunity to analyse the functionality of the EnHANCE collaboration and operations to inform ways of working in the subsequent Round 6. The primary aim of this study was to undertake a critical reflection with the EnHANCE stakeholders, to understand the successes and challenges of participating in this partnership from an individual and organisational perspective. The secondary aim was to identify actionable measures that can be implemented to improve the collaboration in the implementation of the EnHANCE research group in the PRSP (Round 6) funding.

## 2 Methods

This research was reviewed and approved by the Greater Western Human Research Ethics Committee 2022/ETH01283.

### 2.1 Participant Recruitment

Thirty stakeholders involved in EnHANCE projects funded by the PRSP were invited to participate in an anonymous online survey. To be eligible for inclusion, participants had to be actively involved in one or more of the PRSP (Round 5) projects funded to UOW, as either a NSW Health employee, UOW staff member, or student, and to have attended the monthly working groups or governance meetings for a period of greater than 2 months.

### 2.2 Study Design and Theoretical Framework

This qualitative reflective study utilised an open‐ended survey approach based on the Gibbs Reflective Framework [6]. The Gibbs Reflective Framework is a cyclical model that provides individuals an opportunity to reflect on workplace situations, understand successful elements, and identify potential improvements [6]. The cycle consists of six stages, and the survey was structured to align with these stages and designed to:

- Reflect on the perceived roles and responsibilities of different stakeholders.
- Understand the collaboration's successes over the previous 5 years.
- Identify any challenges encountered during project implementation.
- Explore potential improvements for future collaborative efforts.

The survey was created using Qualtrics (Qualtrics, Provo, UT) with a Phonic extension, allowing participants two response options: standard typed responses and voice recording with voice‐to‐text functionality. Qualtrics has a 20 000 character limit per response for open‐ended questions, with Phonic applying this same limit. No time limit was placed on voice recordings. Participants were given 4 weeks to complete their responses, with the ability to save and edit their submissions. Anonymity was guaranteed to encourage candid feedback. Participants were asked to nominate their role, which projects they were involved with, and to refrain from using any identifiable information for either themselves or other individuals.

### 2.3 Stage 1: Description

In this initial stage, participants were asked to provide a detailed account of their collaborative research experience. This included identifying the objectives of the research, the roles of team members, and the outcomes achieved.

Survey question 1: *Describe* your experience of working in partnership on the collaborative EnHANCE projects.

○ You may like to think about…
  - Your role
  - What you had hoped to get out of the collaboration
  - Outcomes from the funding and how the working groups (or governance team) functioned

### 2.4 Stage 2: Feelings

Participants reflected on their emotional responses throughout the collaboration. For example, this could have involved feelings of excitement when ideas were shared or frustration during conflicts.

Survey question 2: How did you *feel* when working in partnership on the collaborative EnHANCE projects?

○ You may like to think about…
  - How you felt you contributed to the outcomes
  - How being part of EnHANCE added value to how you perform your role (either within EnHANCE or within your larger organisation)
  - Whether you were valued as a stakeholder and had your voice heard
  - How you felt about attending the regular meetings

### 2.5 Stage 3: Evaluation

This stage involved assessing what went well and what did not. Successes may have included effective teamwork, innovative solutions, or achieving research milestones, while challenges could have involved miscommunication or unequal participation among team members.

Question 3: What is your *evaluation* of the experience, both good and bad?

○ You may like to think about…
  - What was good and bad about the collaboration?
  - What went well?
  - What didn't go so well?
  - What did you and other people contribute to the collaboration (positively or negatively)?

### 2.6 Stage 4: Analysis

In this stage, participants were asked to delve deeper into the reasons behind successes and challenges. Analysing why certain strategies worked or failed can reveal insights about team dynamics, leadership styles, or external factors affecting collaboration.

Survey question 4: What is your *understanding* about why things went well or didn't go well?

○ You may like to think about…
  - Any real or perceived power imbalances; any structural or functional issues; any differences in organisational priorities; any differences in desired end goals; and how these were/were not overcome

### 2.7 Stage 5: Conclusion

Based on the evaluation and analysis, participants were asked to draw conclusions about their experiences. This included insights into personal strengths and weaknesses in collaboration or broader lessons about effective teamwork practices that could benefit future projects.

Survey question 5: What have you learned through working in partnership on the collaborative EnHANCE projects and what *could have been done differently*?

○ You may like to think about…
  - Your reflections and highlight what changes to your/others' actions could improve the future outcomes
  - What skills do I or others need to develop to more actively contribute to the EnHANCE projects funded through the PRSP (Round 6)?

### 2.8 Stage 6: Action Plan

Participants were asked to develop an action plan outlining steps to enhance future collaborative efforts. For example, this could involve setting clearer communication protocols or establishing roles more effectively.

Survey question 6: What *will you do differently* either as an individual or organisation to maximise the benefits of collaboration to meet the goals of the EnHANCE projects funded by the PRSP (Round 6)?

○ You may like to think about…
  - Moving forward what will you do differently; how will you develop the required skills you need?

## 3 Data Analysis

Qualitative thematic analysis of the stakeholders' responses was conducted separately by the two authors using a deductive, open coding process. This involved immersion in the data and manually assigning codes to meaningful quotes and subsequent grouping into sub‐themes. These aligned with the following four areas: roles and responsibilities, successes, challenges and strategies that can be developed to enhance inter‐organisational collaboration. The authors compared their analyses and any differences in categorisation were settled through discussions between the two authors.

The findings are presented together with a discussion of relevant literature as suggested by Blignault and Ritchie, with each subheading representing the emergent themes [7].

## 4 Results and Discussion

### 4.1 Sample Characteristics and Perceived Roles and Responsibilities

Fifteen participants responded, representing a diverse group including UOW academics, PhD candidates, and NSW Health staff from the Centre for Population Health and at the local health district level (including managers and health promotion officers).

NSW Health staff perceived their primary responsibilities as providing expertise on research translation, aligning research with current health priorities, and offering insights into real‐world implementation constraints. One health employee noted:

My role was to advise on aspects of the research, contribute to discussion and reflection and to provide advice and expertise on the translation of the research to the health jurisdictions. (Participant 15, NSW Health manager).

Conversely, university researchers identified their roles as learning about real‐world research implications, gaining insights into local health system functioning, and understanding program planning processes. An academic remarked:

I have also gained great insights into local health jurisdiction functioning, program planning processes and constraints of implementing research in a real‐world environment. (Participant 12, UOW academic).

### 4.2 Collaborative Challenges

#### 4.2.1 COVID‐19 Disruption

Several significant challenges emerged during the collaborative process. The COVID‐19 pandemic notably disrupted research efforts, with NSW Health staff redeployed to pandemic response and reduced representation at state‐wide policy levels from March 2020 to December 2021.

I guess one thing that has been a bit tricky about the collaboration has been the change of staff at CPH [policy level] over the course of the funding [in part due to pandemic redeployment]. While this has been outside the organisation's control it will be great moving forward if there will be a consistent person attending EnHANCE meetings and being involved in the projects. (Participant 01 NSW Health manager)

Unsurprisingly, these disruptions were experienced right across the health sector, causing delays and difficulties in non‐COVID related research initiatives [8, 9]. Staff redeployment and reduced policy‐level representation underscored the need for flexible, resilient collaborative models that can adapt to unexpected circumstances.

Some research needed to be delayed or a different method of data collection or intervention implementation had to be applied. Over the course of a couple of projects, there had also been turnover of staff which at times led to time needed to orientate new staff to projects. (Participant 12, UOW academic)

#### 4.2.2 Organisational Priorities and Alignment

Jurisdictional diversity presented another complexity, with researchers navigating priorities across multiple health jurisdictions and having to balance the focus on state‐wide scalability with individual jurisdiction needs. Challenges in conducting cross‐jurisdictional research are a common phenomena [10]. While working across multiple contexts complicated research implementation, it also provided a unique opportunity for understanding context‐specific nuances in health promotion strategies.

The LHDs involved are quite different and sometimes having to think about what the outcomes for the LHD were and also consider the outcomes for the other LHD could be challenging. Our LHD is quite diverse so ensuring equity is sometimes difficult. I think everyone involved was able to contribute effectively and all sides were listened to and reflection occurred. (Participant 15, NSW Health manager).

Initial misalignment between research and health priorities evolved over time to a more integrated approach to goal setting, acknowledging the importance of localised, adaptive approaches to population health interventions [4].

I thought it also gave the research some clearer perspective when the researchers were more understanding of the conditions of working on the ground. It enabled troubleshooting and clearer expectations of what the research was actually going to achieve. (Participant 15, NSW Health manager).

The gradual alignment of research goals with both NSW Health and researcher priorities demonstrates the potential for iterative, responsive research partnerships. This approach and findings are echoed in the National Health and Medical Research Council accredited Australian Health Research Alliance model which supports Research Translation Centres comprising health services connected to research institutes and/or universities across Australia [11], and reinforces that such partnerships not only align research goals across organisations but also enhance responsiveness to emerging health challenges in the community [12].

### 4.3 Collaborative Successes

#### 4.3.1 Mutually Beneficial Partnerships

The collaboration saw several notable successes, including the establishment of a strong, equitable, and reciprocal partnership. Relationships strengthened over the five‐year funding period, which enabled timely local implementation of research findings and co‐development of intervention ideas responsive to both state and local contexts. Health Promotion staff gained opportunities for cross‐jurisdictional work, broadening their perspectives and enhancing understanding of research implementation across different settings, as indicated by the following:

The research collaboration has been very beneficial to my role and has allowed me to make valuable contributions to projects that relate to and affect my work. (Participant 02, health promotion officer).

It was a truly collaborative role, with opportunities to shape the research from the start to inform meaningful outcomes and shape future ways of working at NSW Health… In addition to the research findings informing future work direction, I had also hoped to refine my understanding of research methods and processes. Through my involvement in the partnership I was able to do this (Participant 10, NSW Health manager)

Such collaborative successes echo the principles of the research‐practice partnership approach, which emphasise long‐term relationships built on trust and mutual benefit [13].

#### 4.3.2 Fast Knowledge Translation

One notable outcome was the ability to quickly translate research findings into local practice. Traditional research models often delay the application of findings; however, this collaborative approach facilitated immediate implementation even before formal publication. This dynamic model represents a shift towards more effective knowledge translation practices. Such immediate impacts are consistent with findings from research‐practice partnership literature that highlight how these partnerships can accelerate the application of research in practice settings [13].

The research has enabled me to have a better and clearer understanding of the setting and the impacts on the setting to achieve HEAL [healthy eating and active living] related activities across my LHD. I have been able to use this information effectively within my Service and at a broader strategic level with planning. (Participant 15, NSW Health manager).

#### 4.3.3 Reciprocal Learning and Expertise

The study also highlighted reciprocal learning benefits. Health Promotion Officers gained opportunities to work across jurisdictions, broadening their perspective on research implementation.

Working in partnership with the ENHANCE group does allow us to see perspectives from different LHDs as well (e.g., SWSLHD vs ISLHD). (Participant 03, health promotion officer).

Simultaneously, researchers developed deeper insights into real‐world constraints and local health system functioning.

Collaboration between various stakeholders definitely adds value and enriches research being done. It puts a practical face on research and creates a bridge between research and policy/practice. (Participant 05, PhD candidate).

This mutual exchange of expertise challenges traditional hierarchical models of research and practice, with one UOW student commenting:

Sharing knowledge should go both ways. UOW can help Health, just as they help us. (Participant 05, PhD candidate).

The concept of reciprocal learning is well‐documented in collaborative frameworks, emphasising that mutual learning enhances both practice and research outcomes [14].

#### 4.3.4 Adaptive Project Leadership

Many stakeholders highlighted that a key element to the collaboration's success was the strong project governance and management. Monthly meetings provided scheduled opportunities for planning, project updates, discussion and problem‐solving. A consistent time in attendees' calendars, with a clear meeting structure and timely distribution of agendas, ensured regular attendance from project stakeholders.

The EnHANCE project has been really well organised, particularly the ongoing regular meetings which provide timely opportunities for sharing updates and gathering inputs. (Participant 11, CPH, policy‐maker).

What has worked well is the ongoing commitment of LHD staff, to both attend meetings and engage in follow‐up actions (including providing feedback and relevant inputs). (Participant 12, UOW academic).

Initially, there were perceived power imbalances in these meetings, with some UOW academics feeling that perhaps NSW Health staff (particularly health promotion officers) did not have the confidence to speak. In addition, it was noted that meetings could be more forward‐looking rather than focusing on past achievements. This led to a structural change where monthly updates were produced, disseminated along with the agenda with clear questions on discussion points and background information. These were ‘taken as read’, allowing PhD candidates and the research team to provide LHD staff with advance notice of upcoming questions and enabling more time for reflection and opportunity for strategic discussions.

The monthly meetings have improved and after some changes, it seems there is more rich discussions and productive meetings. (Participant 09, PhD candidate).

This reflection on power imbalances, which occurs in collaborative efforts [15], prompted new approaches to working, communicating and planning. The team moved away from traditional ways of drafting manuscripts, creating space for discussions where results were presented at meetings, and findings were explored in detail. This approach ensured the working group was firstly immersed in the process, and secondly had both the space and confidence to contribute based on their expertise, evidenced by the following cited publications [16, 17, 18, 19, 20, 21, 22, 23, 24, 25, 26, 27, 28, 29, 30, 31, 32, 33, 34].

These examples of sound governance and project management exemplify good research practice, particularly when working collaboratively, resulting in streamlined processes, enhanced efficiency, and high‐quality research outcomes [35, 36].

## 5 Methodological Reflections

The use of Gibbs' Reflective Cycle provided a structured yet flexible approach to understanding collaborative dynamics [6]. By creating space for critical reflection, the methodology allowed stakeholders to articulate both successes and challenges candidly. This approach itself represented a useful method for evaluating inter‐organisational research partnerships.

## 6 Implications for Future Collaborations

The Prevention Research Support Program model offers a promising framework for future collaborative research. Key implications of this funding scheme include the opportunity, time, and funding to create the sustained long‐term partnerships.

The greatest benefit was for our LHD to be formally linked in research activities with academics over a significant period of time. This allowed the staff of both organisations to develop positive and productive working relationships. (Participant 08, NSW Health manager).

This sustained partnership facilitates pathways for ongoing alignment of priorities, ensuring the needs of future evidence development adapt as priorities change and in response to everchanging contexts; for example, change in government leadership or organisational priorities and funding. Finally, this funding scheme, when implemented effectively, has the opportunity to strengthen two‐way communication between researchers and practitioners/policy makers and value and harness the expertise of all collaborators whilst providing mutually beneficial outcomes [37].

## 7 Limitations

While the study provides valuable insights, limitations include the relatively small sample size (*n* = 15) and the focus on a state‐level health system and a specific research group within one university. The findings may not be directly generalisable to all research contexts, though they offer important transferable lessons beyond community‐based health promotion and healthy eating and active living settings.

## 8 Conclusion

The PRSP demonstrates an effective funding model for facilitating meaningful collaboration between researchers, policymakers, and practitioners, specifically under the EnHANCE research group. By creating space for shared goal‐setting, mutual learning, and flexible research translation, the PRSP funding program has contributed to developing a more integrated approach to promoting healthy eating and active living for children. Future research should continue to explore and refine collaborative models that bridge traditional institutional boundaries, focusing on creating adaptive, responsive systems of knowledge generation and implementation. Furthermore, we should ensure as researchers that we take a reflexive approach to our practice with the aim of developing more resilient, productive inter‐organisational research partnerships.

## Author Contributions

**Sarah T. Ryan:** conceptualisation, methodology, formal analysis, investigation, project administration, writing – original draft. **Jennifer Norman:** conceptualisation, methodology, formal analysis, investigation, writing – original draft.

## Ethics Statement

This study was conducted following the ethical guidelines and principles outlined by The University of Wollongong Health and Medical institutional review board: HREC 2022/ETH01283.

## Conflicts of Interest

The authors declare no conflicts of interest.

## Data Availability

The data that support the findings of this study are available from the corresponding author upon reasonable request.
